# Mapping of the Gastrointestinal Short Form Questionnaire (GSF-Q) into EQ-5D-3L and SF-6D in patients with gastroesophageal reflux disease

**DOI:** 10.1186/s12955-018-1003-y

**Published:** 2018-09-10

**Authors:** Manuel Monroy, Miguel A. Ruiz, Javier Rejas, Javier Soto

**Affiliations:** 1 Faculty of Psychology, Universidad Autónoma de Madrid, C/ Ivan Pavlov 6, 28049 Madrid, Spain; 2 Faculty of Economics, Universidad Calos III de Madrid, C/ Madrid, 126, 28903 Getafe, Madrid, Spain

## Abstract

**Background:**

The short, self-administered Gastroesophageal Reflux Disease (GERD) Symptom Frequency Questionnaire (GSFQ) is a specific Quality of Life (QoL) instrument which measures the impact of GERD symptoms on QoL. This study aims to map the specific scores in GSFQ into two generic instruments: SF-6D and EQ-5D-3 L, in order to obtain utility estimates derived from the GERD condition.

**Method:**

A national representative sample of GERD patients was selected, stratified by gender, age (< 45, ≥45 years) and GERD severity (0-I, II-IV Savary-Miller score) for validation purposes. Age, gender, BMI, GERD diagnose, GERD severity, associated comorbidities and risk factors were recorded. GSFQ, SF-6D, EQ-5D-3 L, and the HRQoL Visual Analogue Scale (VAS) were answered by patients. Several mapping methods were estimated, regression using dummy variables, and linear, quadratic and cubic regression using optimal factor scores. The use of a GERD aggregated summary severity derived from the GSFQ was dimed the best predictor. Overall Mean Absolute Error (MAE), overall Mean Absolute Percentage Error (MAPE) were used as goodness-of-fit (GOF) indexes to compare models.

**Results:**

A total of 3405 patients were recruited by 490 clinicians. Mean age was 49 (±14.4) years and 49.8% were women. Reported comorbidities were clustered in 6 antecedents and 15 concomitant pathologies. Aggregation of levels for the frequency of symptoms items was found more suitable for estimation. Regression weights were found to follow a monotonous progressive pattern. Overall MAE ranged from 0.092 to 0.094 for SF-6D utility prediction and from 0.008 to 0.08 for EQ-5D-3 L, while MAPE values ranged from 27.9 to 29% for SF-6D and from 36.8 to 38.4% for EQ-5D-3 L. Cubic regression GOF demonstrated a better fit.

**Conclusions:**

It is possible to translate specific GSFQ scores assessing GERD condition into generic SF-6D and EQ-5D-3 L utility values. Although regression using dummy variables is a suitable mapping procedure, other alternative mapping methods convey better fit, in particular cubic regression.

## Background

Gastroesophageal Reflux Disease (GERD) appears when stomach contents flux back to the esophagus. It happens when the valve located between the esophagus and the stomach does not close properly. Most frequent disease symptoms are acidity and acid reflux. Other less frequent but associated symptoms are heartburn without clear motive, panting, throat ache and cough, among others [1, 2].

GERD can be classified into four severity levels, ranging from the appearance of edema and erythema, causing some degree of esophagus erosion, up to esophageal ulcers or Barret’s esophagus. Consumption of alcohol or carbonated drinks, obesity and smoking are known to be GERD risk factors [3].

According to the DIGEST international study, approximately 7.7% of the population suffers from GERD [4]. Attending to the current consensual definition: “GERD should be used to include all individuals who are exposed to the risk of physical complications from gastroesophageal reflux, or who experience clinically significant impairment of health related well-being (quality of life) due to reflux related symptoms, after adequate reassurance of the benign nature of their symptoms” [5]. Furthermore, it is commonly accepted that self-reporting is one of the main sources of diagnosis [6] and patients should report experiencing symptoms at least twice a week [2, 7] for a diagnosis of GERD.

It is important to remark that the impairments caused by GERD symptoms are highly variable and may affect quality of life even when there are no endoscopic findings [2]. Patients tend to adopt eating behaviors in order to prevent or attenuate their clinical situation. The Agency for Healthcare Research and QALY reports that the more frequent treatments are antacids (neutralizing stomach acids) and type 2 histamine receptor antagonists (H_2_RA) or proton pump inhibitors (PPI), both reducing the production of stomach acid [8, 9]. The impacts of GERD symptoms on patients’ health-related quality of life (HRQoL) is usually ascertained by means of patient-reported-outcomes measurements (PROMs) such as the Gastrointestinal Short Form Questionnaire (GSF-Q) [10].

HRQoL measures are particularly important for GERD sufferers given their diagnostic capabilities, while they also reveal important issues to health service providers for several reasons. First, HRQoL has been shown to have a direct relation with mortality, hospitalization and consumption of clinical resources. Second, it has been shown to have a low to moderate relation with other disease-specific indicators, hence contributing complementary information for assessing clinical impairment [11]. Presently, HRQoL has been identified as a clinical target in itself, both in patients with limited life expectancy and for therapies directed towards disease coping or symptom accommodation, as much as for biological improvement (as is the case for most chronic diseases). Preference-based measures (PBMs) play a central role in these evaluations. They allow patients to describe the impact of ill health and have an associated “utility” score for each health-state description. These utility scores can then be used to calculate quality-adjusted life-years (QALYs), which is an outcome metric used in many economic evaluations of potential health benefits [12].

In the past, clinical studies did not always include a PBM. Often they included one or more of the many PROMs that are not full PBMs because they do not have an associated, preference-based scoring system. On the other hand, PROMs have proved to be very sensitive to variations in patient health conditions, and this is one of the reasons for their extended use in clinical studies. Furthermore, when a major research need is to compare result with those of other pathologies or comorbidities, it will not be possible to use disease-specific PROMs, and generic HRQoL instruments should be preferred. Most popular generic instruments (like SF-6D, EQ-5D and HUI3), offer the possibility of computing the utility score associated to each health condition (as captured by the instrument attribute profile), reflecting the population preference towards each health state in a situation of uncertainty. This peculiarity allows using them in computing QALYs and in health economics in general.

It is usually the case that a disease-specific PROM instrument will be preferred in research about a particular disorder and when the use of generic instruments has been avoided because they do not capture properly the different levels of disease symptomatology on patients’ HRQoL. Also, because there is evidence suggesting that generic measurements might have poor sensitivity to change in some health conditions, such as GERD or others non-threatening illnesses, or are incapable of discriminating well between patients using different drugs to treat their health problems [13, 14]. In such cases, the usual strategy is to map the specific measurements into a generic instrument allowing further comparison with other studies in which the specific instruments may not be pertinent or are otherwise unavailable (e.g., retrospective databases) [15, 16].

Aligned with such an approach, since 2008, NICE’s preferred measure of health-related quality of life in adults has been EQ-5D, to derive utilities set values for health economic evaluations (see Guide to the methods of technology appraisal 2013, at https://www.nice.org.uk/process/pmg9/chapter/foreword.

The aim of the present study was to obtain the mapping algorithms needed for translating the specific HRQoL measure obtained by the GSF-Q into two of the most popular preference-based generic instruments, the SF-6D and the EQ-5D-3 L. As a secondary benefit, we will be able to assess which one of the generic instruments is more suitable for capturing HRQoL deterioration due to GERD conditions.

## Methods

### Study design

The present study is a secondary analysis carried out using the data gathered for the cultural validation of the GSF-Q into Spanish [17]. The original study was developed to ensure adequate estimation of psychometric properties, and was designed as an observational study that would provide a rich data set, not only for instrument validation but particularly for mapping studies, beyond what could be obtained in controlled clinical trials. This was a cross-sectional, single time point assessment design. The original sample design was thought to ensure representativeness of three strata: gender, age (< 45, ≥45 years) and symptom severity (Savary-Miller: 0-I, ≥II). Patients were selected at random by demand of attention and covering each sample stratum. Scales were administered in a single visit. Patients were over 18 years of age, able to read Spanish, and signed an informed consent form. The Ethics Committee of one of the participating centers in the validation study was responsible for approving the study design. Clinicians were recruited at random and proportionally on the geographical extension and service demand in the Spanish Autonomous Communities. The study recruited the participation of 510 gastroenterologists, and they were requested to provide 4 to 8 subjects each. Additional data on the study design may be found elsewhere [17].

### Participants

The final sample was composed by 3405 patients, from whom 2251 completed all the questionnaires, sociodemographic and clinical data. Half of the participants were women (49.8%), 63.9% were obese, 40.1% smokers, 42.8% consumed alcohol, and 46.5% consumed carbonated beverages. GERD was diagnosed in 80% of cases, 46.3% were under IBP treatment, 16.5% used H_2_RA, and 25.3% used antacids. It should be mentioned that 48.4% were on treatment for at least one other comorbidity (Table 1). All patients had signed informed consent forms, and the Helsinki declaration guidelines were met.

**Table 1:** Sample sociodemographic and clinical descriptors

Variable	Level	Frequency	Percent
Age (decades)	18–30	147	6.5
	31–40	392	17.4
	41–50	510	22.7
	51–60	529	23.5
	61–70	431	19.1
	71–80	187	8.3
	> 80	55	2.4
Gender	Male	1131	50.2
	Female	1120	49.8
Smoking	Yes	903	40.1
	No	1348	59.9
Alcohol	Yes	963	42.8
	No	1288	57.2
antiH2	Yes	348	15.5
	No	1903	84.5
Treated for comorbidities	Yes	1161	51.6
	No	1090	48.4
GERD Level	0	396	17.6
	1	521	23.1
	2	583	25.9
	3	220	9.8
	4	102	4.5
	Unknown	429	19.1
Body Mass Index	Infra-weight	17	.8
	Normal	796	35.4
	Over-weight	1438	63.9
Carbonated Drinks	Yes	1047	46.5
	No	1204	53.5
IBP	Yes	965	42.9
	No	1286	57.1
Antacid	Yes	556	24.7
	No	1695	75.3

### Instruments

Three questionnaires were used to measure HRQoL, the 2 most popular generic ones and a GERD specific instrument.

The *Gastrointestinal Short Form Questionnaire* (GSF-Q) [6, 7], was used to measure GERD symptom impact on HRQoL. The questionnaire is composed of six items, plus 2 filter items. The first four gauge the impact of GERD symptoms during the most recent week (upper abdomen pain, breastbone pain, limited eating, heartburn) using a 5-point Likert scale (0 = Never, 4 = All of the time). The last two inquire about the number of days per week with daytime or nighttime disturbances (0–7 days). The total score is obtained by adding up individual item scores, and it is customary to rescale it into a 0–100 severity scale. A higher score represents a higher impact on HRQoL and scores are usually interpreted by comparison with population norms [17].

*EuroQol-5 Dimension-3 Levels* (EQ-5D-3L) [18, 19] is a generic, preference-based HRQoL instrument. It gathers the level of deterioration for 5 attributes: mobility, self-care, usual activities, pain/discomfort, and anxiety/depression; using 3-level items (1 = none, 2 = some problems, 3 = a lot of problems). Each combination of levels creates a health profile, with a total of 243 possible health states, although not all of them are equally likely. Profile [11111] corresponds to perfect health and profile [33333] represents the worse possible health state. Based on population preference ranking, health states are translated to a social utility value using a multi-attribute utility function (MAUF). Different MAUFs are used for different countries, mainly using estimates based on Time Trade-Off (TTO) and Visual Analogue Scale (VAS) methods [20]. The basic form of the EQ-5D-3 L MAUF is:

$$ {u}_i=1-\left(q+\sum \limits_{j=1}^{j=5}\sum \limits_{k=1}^{k=3}{b}_{jk}{D}_{ijk}+{b}_{N 3}N{3}_i\right) $$

Where the utility/preference value for health state *i* (*u*_*i*_) is obtained by subtracting from 1 the health state disutility (${\overline{u}}_i$). Disutility is obtained by weighting (*b*_*jk*_) the deterioration level *k* attained in dimension *D*_*j*_, plus an interaction term (*N*3_*i*_), which adds a constant *b*_*N*3_ when any of the dimensions reaches its maximum deterioration level, plus a constant (*q*). It should be noted that *b*_*j*1_ = 0 for the first level of any dimension (*k* = 1), which represents no deterioration in that dimension [21].

The *Medical Outcomes Survey Sort Form-6 Dimension* (SF-6D) [22, 23] is a generic, preference-based HRQoL instrument derived from the 36-item MOS SF-36 [24]. It gathers the level of deterioration for 6 dimensions: physical functioning, role limitations, social functioning, pain, mental health, and vitality; using a recoding of 11 specific items into 4 to 6 levels. A total of 18,000 health profiles are possible, with the profile [111111] corresponding to perfect health and [645655] representing the worse possible health state. Different MAUFs have been estimated for deriving preference utilities in different countries, with the peculiarity that no severity (interaction) constant is used. As in the previous case, a value of 0 is assigned to the first level for each dimension/attribute.

### Statistical analyses

The first step consisted in checking the unidimensionality of GSF-Q items and, if met, deriving an overall severity index due to GERD condition. This severity index will be used to short generic health states (EQ-5D-3 L or SF-6D) when their corresponding profiles differ only in the permutation of one severity level, e.g.: [11112] vs. [11121]. A first approach was to estimate a unidimensional latent variable model assuming the latent variable to be continuous and items/indicators to be ordinal while using the WLSMV estimation method. A second approach was to decompose each k-categories item into a series of k dummy variables (0 = No, 1 = Yes) and coding lower level dummy categories as fulfilled (1) when a particular item-level was reached. A Partial Credit model [25] (an extension of the Rasch model) using ML estimation was obtained. In this way, estimated category thresholds could be compared across items and monotonic distribution of item step thresholds could be checked. Observed EQ-5D and SF-6D utility mean scores were compared using standard t-test and using bootstrap estimates in order to avoid the influence of skewness and extreme utility values.

Once a summary GERD-specific severity index was obtained, this index was mapped onto each of two utility values (separately), and several models were tested (see below) in order to predict the utility value associated to each GERD severity condition.

Disutility values (*d*_*i*_ = 1-*u*_*i*_) were modeled, instead of utility values, for several reasons. First, the data-mass usually concentrates around more lenient health states, and low disutilities will fall closer to the axis origin. Second, it is always possible to estimate a model without the intercept term, anchoring 0 value disutilities (perfect health) at the 0 GERD severity value. Since GERD is not necessarily a disabling condition, and in order to attenuate the impact of possible comorbidities in the disutility value for each individual, disutilities were aggregated, using the mean value, by GERD severity, before modelling.

The following regression models estimated linear, quadratic and cubic trends, using density function values, and Tobit and Probit, using cumulative distribution values. The following covariates were tested for inclusion: Age (decades), BMI (low, normal and overweight), GERD diagnosis (Yes), smoking, alcohol consumption, carbonated drinks consumption, IBP treatment, H_2_RA treatment, antacid treatment, and treatment for comorbidities. In order to anchor the best possible health states in both instruments, the GERD severity factor scores were rescaled into the range 0–1, and regression models were fit through the origin.

Along with the statistical significance of regression coefficients, model goodness-of-fit (GOF) was assessed using R^2^, mean absolute error (MAE) and mean absolute percentage error (MAPE). MAE and MAPE were computed overall and by quintile group based on severity scores to assess local GOF at the different levels of severity. Bootstrap estimates for model coefficient standard errors were also obtained to avoid the influence of outlier observations in the assessment of parameter significance levels. General internationally-accepted guidelines proposed for instrument mapping were followed [13].

All analyses were conducted using the SPSS for Windows statistical software, version 22.0 and Mplus 7.

## Results

GSF-Q scores ranged between 0 and 30 with a mean value of 10.54 (SD = 5.94). GERD Severity summary scores (factor scores) ranged between − 1.40 and 1.88 with a mean value of 0 (SD = 0.636) with a symmetric distribution (Skewness = 0.021, SE = 0.052).

At the individual level, SF-6D mean utility scores (M_SF_ = 0.656, SD_SF_ = 0.207) were significantly lower than EQ-5D-3 L scores (M_EQ_ = 0.744, SD_EQ_ = 0.206), both under asymptotic assumptions (*t* = − 27.54*, p* < 0.001) and using 10.000 bootstrap samples: Difference 95% CI = (− 0.093, − 0.081), suggesting that slightly higher utilities were obtained with the EQ-5D. As expected, both utility scores showed a marked negative skewness, SF-6D: Skewness_SF_ = − 0.784, SE_SF_ = 0.052; EQ-5D-3 L: Skewness_EQ_ = − 1.049, SE_EQ_ = 0.052, with a high correlation between them (*r* = 0.733, *p* < 0.001).

**Fig. 1:**
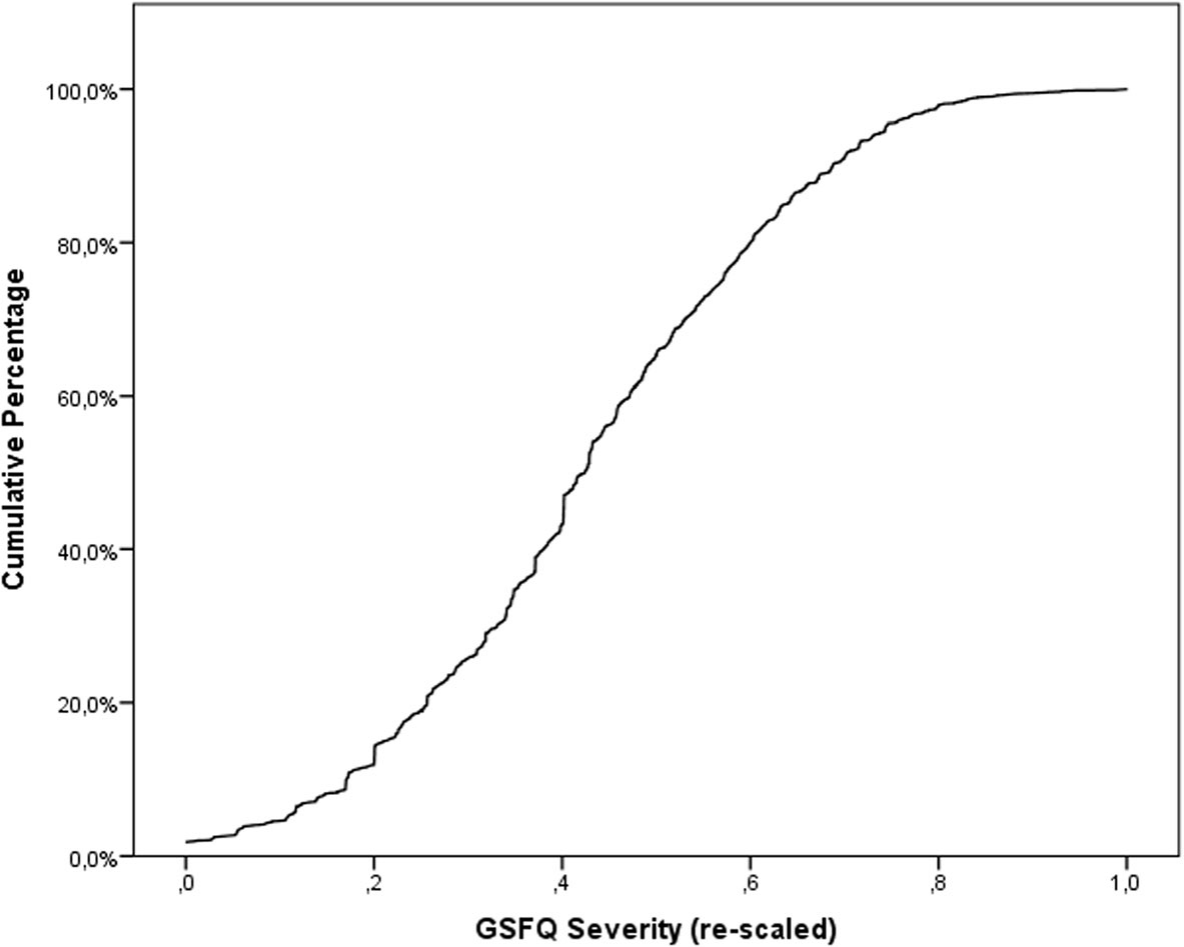
GSF-Q severity score cumulative distribution

**Fig. 2:**
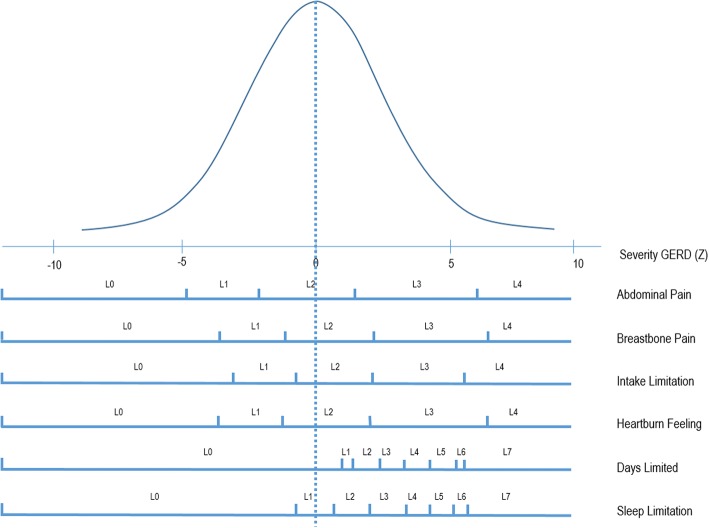
GSF-Q item thresholds assuming unidimensionality (Partial Credit Model)

The first eigenvalue of the correlation matrix was λ = 3.55 and all further eigenvalues were below 1. The confirmatory factor analysis for the 1-dimension solution (assuming variables to be ordinal) attained good GOF indexes with CFI = 0.951 and TLI = 0.918. Figure 1 shows the cumulative distribution for rescaled factor scores, exhibiting a smooth ogive distribution with no evident changes in curvature. This figure may be used as normative data to obtain percentiles from severity scores. Figure 2 represents the response category thresholds for each item with respect to the latent normal severity score. In this figure, severity scores are expressed in standard deviations from the mean latent severity of 0 and, for each GSF-Q item, partial credit thresholds for each step rating response are plotted, showing a rather even spread and separation of rating categories for the first four items, and a displacement of the category thresholds above the mean severity for the last two items of daytime and nighttime limitations. This later result is in accordance with the smaller weight received by the two last items in computing the factor score.

**Fig. 3:**
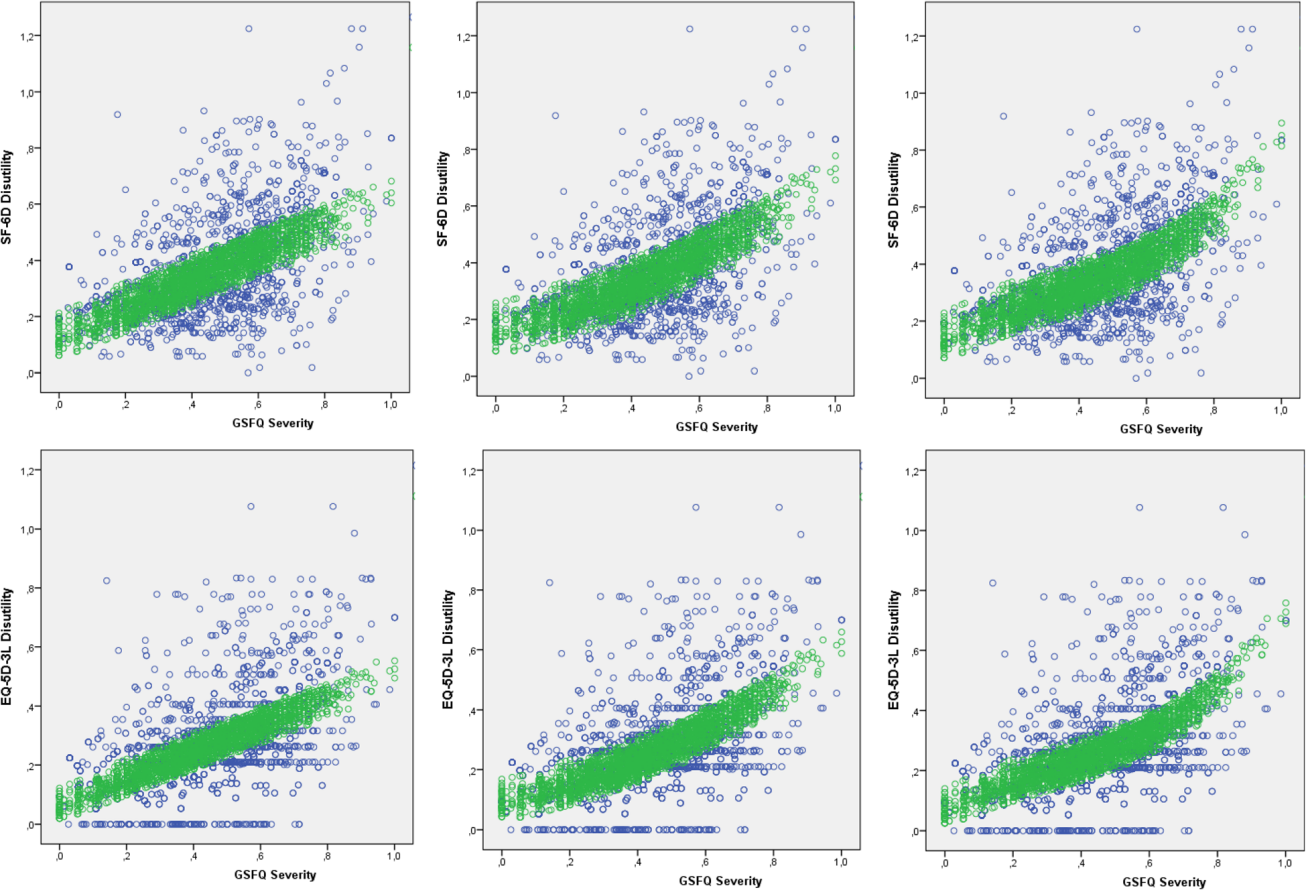
SF-6D: (up) and EQ-5D-3 L (down) observed (blue) and predicted (green) utility values vs. GERD severity for the linear (left), quadratic (center) and cubic (right) models

The resulting equation needed for computing re-scaled estimated factor scores from observed GSF-Q items scores may be expressed as follows:

$$ {\widehat{f}}_i=\left(0.183{x}_{1i}+0.204{x}_{2i}+0.100{x}_{3i}+0.174{x}_{4i}+0.047{x}_{5i}+0.044{x}_{6i}+1.4025\right)\times 0.30479 $$

Where *x*_1_ to *x*_4_ are the scores in the first 4 GSF-Q items (0 = Never, 4 = Always), *x*_5_ is the number of days with disability, *x*_6_ is the number of nights with GERD problems, and 1.4025 and 0.30479 are scaling constants moving the factor scores into the 0–1 range.

EQ-5D-3 L showed to be particularly less sensitive to GERD severity. Only 78 (32%) of the 243 possible EQ-5D-3 L profiles were observed and 17 (7%) of them gathered more than 90% of patients. Table 2 shows the most frequent EQ-5D-3 L profiles observed in our sample. In the case for SF-6D utility scores, 975 (5.4%) out of the 18,000 possible health states were observed, 35 (0.2%) profiles presented a prevalence above 5/1000, gathering only 25.5% of cases.

**Table 2:** Most prevalent EQ-5D-3 L and SF-6D health state profiles, associated utilities, and frequencies (cases, percentages and cumulative percentages; partial listing)

Profile	Utility	Freq.	Percent	Cum. %
EQ-5D				
11111	1.00	566	25.1	25.1
11121	.79	376	16.7	41.8
11122	.74	294	13.1	54.9
11222	.68	166	7.4	62.3
21222	.59	118	5.2	67.5
11112	.80	107	4.8	72.3
11221	.74	99	4.4	76.7
22222	.49	52	2.3	79.0
21221	.65	50	2.2	81.2
21121	.70	36	1.6	82.8
21122	.65	31	1.4	84.2
11223	.42	30	1.3	85.5
22232	.22	30	1.3	86.9
22221	.54	22	1.0	87.8
11232	.41	17	.8	88.6
21232	.32	17	.8	89.3
11233	.36	16	.7	90.0
11211	.79	15	.7	90.7
11123	.48	14	.6	91.3
21233	.27	12	.5	91.9
22233	.17	12	.5	92.4
11113	.54	10	.4	92.8
21111	.76	10	.4	93.3
21223	.33	10	.4	93.7
22223	.23	10	.4	94.2
11212	.74	8	.4	94.5
12222	.58	7	.3	94.8
21112	.71	7	.3	95.2
21131	.43	7	.3	95.5
21231	.37	7	.3	95.8
11231	.46	6	.3	96.0
22332	.17	6	.3	96.3
12221	.63	5	.2	96.5
11131	.52	4	.2	96.7
12121	.69	4	.2	96.9
12233	.26	4	.2	97.1
21132	.38	4	.2	97.2
12223	.32	3	.1	97.4
21211	.71	3	.1	97.5
SF-6D				
111222	.86	52	2.3	2.3
111112	.94	30	1.3	3.6
111223	.86	30	1.3	5.0
111122	.88	29	1.3	6.3
111123	.88	22	1.0	7.2
111322	.84	19	.8	8.1
111111	1.00	18	.8	8.9
111212	.92	18	.8	9.7
111224	.79	18	.8	10.5
211224	.78	17	.8	11.2
212324	.73	17	.8	12.0
112324	.74	16	.7	12.7
211222	.84	16	.7	13.4
111323	.84	15	.7	14.1
211323	.83	15	.7	14.7
111225	.76	14	.6	15.4
111324	.78	14	.6	16.0
113424	.55	14	.6	16.6
211324	.76	14	.6	17.2
212323	.79	14	.6	17.9
212325	.69	14	.6	18.5
312323	.77	14	.6	19.1
111121	.93	13	.6	19.7
112322	.80	13	.6	20.3
112323	.80	13	.6	20.8
113324	.72	12	.5	21.4
211223	.84	12	.5	21.9
212322	.79	11	.5	22.4
111221	.92	10	.4	22.8
111325	.74	10	.4	23.3
112222	.82	10	.4	23.7
211322	.83	10	.4	24.2
212224	.74	10	.4	24.6
311324	.75	10	.4	25.1
312324	.71	10	.4	25.5
111124	.81	9	.4	25.9
112122	.84	9	.4	26.3
113323	.78	9	.4	26.7
212223	.81	9	.4	27.1

The best fitting model for mapping GSF-Q into SF-6D disutilities was a cubic model including variables GERD severity (linear, quadratic and cubic), age (in decades), gender, BMI group (infra, normal, and over-weight), and being treated for comorbidities (see Table 3). The model GOF was good (*R*^2^ = 0.888), with MAE = 0.092 and MAPE = 27.9% (Table 4) Fig. 3.

**Table 3:** Estimated model coefficients

Model	Predictor	SF-6D disutility				EQ-5D-3 L disutility			
		B	SE	Beta	Sig	B	SE	Beta	Sig
Linear	GSF-Q severity	.481	.014	.592	<.001	.441	.011	.688	<.001
	Age (decade)	.012	.002	.124	<.001	.010	.002	.132	<.001
	Gender (Female)	.041	.005	.077	<.001	.027	.005	.064	<.001
	BMI (Grouped)	.019	.006	.080	<.001	–	–	–	ns
	Comorbidities (Treated)	.043	.003	.132	<.001	.034	.006	.082	<.001
Quadratic	GSF-Q severity	.241	.044	.297	<.001	.195	.042	.304	<.001
	GSF-Q severity (square)	.291	.051	.214	<.001	.309	.049	.288	<.001
	Age (decade)	.013	.002	.138	<.001	.013	.002	.171	<.001
	Gender (Female)	.045	.005	.084	<.001	.032	.005	.076	<.001
	BMI (Grouped)	.031	.004	.217	<.001	.009	.004	.078	.020
	Comorbidities (Treated)	.040	.006	.075	<.001	.031	.006	.074	<.001
Cubic	GSF-Q severity	.610	.091	.751	<.001	.527	.170	.485	<.001
	GSF-Q severity (square)	−.822	.245	−.605	.001	−.665	.207	−.620	.001
	GSF-Q severity (cube)	.891	.192	.444	<.001	.768	.068	.823	<.001
	Age (decade)	.012	.002	.128	<.001	.013	.002	.167	<.001
	Gender (Female)	.043	.005	.080	<.001	.030	.005	.071	<.001
	BMI (Grouped)	.041	.006	.078	<.001	–	–	–	ns
	Comorbidities (Treated)	.023	.004	.163	<.001	.032	.006	.077	<.001

*ns* not significant

**Table 4:** Estimated model goodness of fit statistics

Model	Overall		MAE						MAPE (%)					
	R^2^	Adj. R^2^	Overall	Q1	Q2	Q3	Q4	Q5	Overall	Q1	Q2	Q3	Q4	Q5
SF-6D														
Linear	.885	.885	.094	.067	.076	.078	.123	.127	29.0	39.0	26.1	23.0	30.8	26.2
Quadratic	.887	.886	.093	.065	.075	.069	.122	.126	28.1	33.4	27.2	23.6	31.3	25.2
Cubic	.888	.887	.092	.065	.075	.069	.120	.125	27.9	33.6	26.0	23.3	31.4	25.2
EQ-5D-3 L														
Linear	.827	.826	.008	.065	.076	.073	.101	.124	38.4	62.3	37.0	28.7	32.6	32.0
Quadratic	.830	.829	.086	.065	.076	.069	.099	.121	36.8	52.2	40.0	29.0	32.8	30.2
Cubic	.831	.831	.086	.065	.076	.076	.098	.119	37.0	33.6	38.2	28.9	33.1	29.8

*MAE* Mean Absolute Error, *MAPE* Mean absolute percentage Error, *Q1-Q5* quintile groups

The best fitting model form mapping GSF-Q onto EQ-5D-3 L disutilities was the cubic model including GERD severity (linear, quadratic and cubic), age (in decades), gender, and being treated for comorbidities. BMI group was not significant and the following GOF statistics were obtained: *R*^2^ = 0.831, MAE = 0.086 and MAPE = 37.0%.

## Discussion

Specific HRQoL instruments are the preferred choice for measuring patient perceptions on their health condition because of their high sensitivity to changes due to disease management and treatment suitability. However, mapping specific HRQoL into generic utility scores can present methodological problems. Albeit the good psychometric properties of instruments like GSF-Q for measuring the impact of GERD on patients’ daily lives [10, 17, 26], GERD is a relatively mild health disabling disease, as compared to other possible health states measured by generic instruments. Besides, it is difficult to instruct patients to restrict their thinking to only one specific disease-related disability, isolating their judgments from other comorbidities that might be present, or from the impact of normal disabilities associated with to aging, when responding to generic instruments. The final result is that generic instruments might capture the effects of other disabilities and limitations which are not be directly related to the specific disease being mapped.

One possible strategy for avoiding these problems would be to design a preference-choice experiment with the health conditions vignettes derived from the specific instrument [27]. Unfortunately, it could be expected that marginal disutilities could be oversized if other, very severe health conditions are included as anchoring. Another possibility could be to describe specific health conditions only by the set of generic health profiles that are prevalent and meaningful in the particular disease, and only mapping those conditions. This approach could be used when observed distributions like the one obtained for the EQ-5D-3 L are found (see Table 2), and a reduced number of health states gather the majority of patients. But, very large samples would need to be used, if the intent is to obtain representative results, and it could be cumbersome when the number of possible health states is very large, as has happened with the SF-6D (Table 2).

In the time being, directly mapping specific health states onto generic utility values seems to remain the best option, and special care should be taken, by aggregating generic utility values over specific severity scores, in order to smooth out the impact of non-specific effects on the mapping estimates. The present paper reports the first study mapping GSF-Q onto two of the most widely used generic HRQoL instruments. In fact, our study could be considered to have high ecological validity due to the large sample used and its ample representativeness.

In our study, GERD was found to be a quite lenient pathology, with mean utility values of 0.656 (SF-6D) and 0.744 (EQ-5D-3 L). In fact, the most prevalent health-attribute level reported was the first (no deterioration), in both generic instruments, except for the attributes/dimensions of pain and Mental Health (see Table 5). Even the scaling of the response levels of one’s own GSF-Q suggests that the third response level (L2 in Fig. 2) had been selected by patients in order to be located above the mean in the latent (error-free) severity score for all items, except for the number of days with problems. These results are in agreement with regular GERD diagnosis, which states that stomach problems should be present more than 2 days a week in order to be consistent with GERD [7].

**Table 5:** Percentage of responses by dimension level for each dimension/attribute of the EQ-5D-3 L and SF-6D generic instruments

Dimension	EQ-5D-3 L			Dimension	SF-6D					
	Dimension Level				Dimension Level					
	1	2	3		1	2	3	4	5	6
Mobility	78.4%	21.2%	0.4%	Physical Function	35.6%	27.8%	22.2%	2.5%.	10.5%	1.4%
Self-care	91.5%	8.1%	0.4%	Role Limitation	54.9%	12.2%	18.3%	19.6%	*	*
Daily activities	66.0%	32.7%	1.3%	Social Function	36.4%	28.7%	27.1%	7.0%	0.8%	*
Pain	32.8%	59.9%	7.3%	Pain	12.3%	19.5%	34.8%	18.7%	13.2%	1.4%
Anxiety/Depression	54.6%	39.1%	6.3%	Mental Health	10.8%	61.8%	17.6%	7.8%	2%	*
				Vitality	5.3%	19.3%	22.8%	28.9%	16.9%	6.8%

* Unused dimension level

Obtained SF-6D utility scores were shown to be more sensitive to GERD-severity than those obtained from EQ-5D-3 L.The distribution of the former was more spread out, with less likelihood of ceiling effects, and did not exhibit a gap between perfect health, *u*(11111) = 1, and the following larger value, as it was the case with the later, *u*(11121) = 0.79. The observed cumulative distribution function of SF-6D disutility scores was more uniform; the distribution function of EQ-5D-3 L disutilities was steeper (especially in the milder health states) and the distributions did not cross over within their ranges.

GSF-Q scores showed good unidimensional behavior which allowed summarization of GERD-related severity in a single score using factor analysis weights. Unidimensionality analyses endorsed the possibility of summarizing the different GERD symptoms in an aggregated overall score, also obtaining an adequate scaling of response levels. In our case, this strategy should be preferred against one using item-response dummy coding in the regression models, since it avoids deciding how to aggregate item response levels [28] and minimizes the possible impact of covariates in particular response levels.

For each of the generic instruments, the best-fitting model was selected. In both cases, the model including GSF-Q severity (observed, squared and cubed), age, gender, and being treated for comorbidities attained the best fit, and the SF-6D model additionally included BMI. The sign of the regression coefficients were in accordance with predicting a higher disutility as GSF-Q severity scores increase. The inclusion of significant covariates by all models suggests that the loss in HRQoL may be influenced not only by GERD symptoms but also by personal comorbidities present. This is to say that GERD symptoms may be not very prominent when assessing HRQoL using a generic instrument if other health conditions might be present, such as aging, being treated for comorbidities and overweight.

*R*^2^ values were within the range 0.885–0.888 for model SF-6D, and within 0.827–0.831 for model EQ-5D-3 L. Overall MAPE = 27.9% for predicting SF-6D and MAPE = 37.0% for predicting EQ-5D-3 L when using predictions derived from the cubic model. Computing predicted SF-6D disutility MAPE by GSF-Q severity quintile groups, MAPE ranged between 33.6% for Q1 and 23.3% for Q3 while for predicted EQ-5D-3 L disutility, MAPE ranged between 33.6% for Q1 and 28.9% for Q3 (see Table 4). As expected, the error magnitude was smaller near the location of the centroid; while it was particularly high when predicting EQ-5D-3 L disutilities using the linear model (up to 62.3% in Q1).

Some additional covariates, like smoking and drinking carbonated beverages or alcohol, approached statistical significance, but all models were kept as parsimonious as possible, and only statically-significant predictors were included (*p* < 0.05). Bootstrap estimates were generated, based on 1000 samples with replacement, obtaining parameter estimate bias smaller than |0.002| and significance levels$\widehat{p}\le 0.002$.

Mapping disease-specific instruments onto generic health related measures is a common methodological strategy due to the high sensitivity of specific instruments and the wide generalizability of generic measures. Mapping the GERD-specific GSF-Q scores onto generic utilities (SF-6D and EQ-5D-3 L) was shown to be possible, attaining adequate goodness-of-fit values. In both cases, the best-fitting model was the more complex one; the model based on GSF-Q severity, raised to the cubic power, and including generic covariates: age, gender, BMI and treatment for comorbidities. However, the model for predicting EQ-5D-3 L disutilities did not include BMI as a statistically significant covariate.

The use of cubic prediction models needs special care, since small variations in the cubed predictors can entail excessively large predicted values, including those for predictors out of the range of the observed data used for prediction, that can produce unreasonable predictions. In our case this prevention is needless, given that all GERD severity values are scaled within the 0–1 range (any value will have to be inside the range of values used for estimation), and possible covariate values are limited to the observed repertoire.

In our study, we found that utility values associated with GERD-specific conditions were rather high, suggesting that this disease is not very disabling (in general). Nevertheless, patients with utility values as low as SF-6D = − 0.3150 and EQ-5D-3 L = − 0.0757 were observed, although they were not always associated with the worst GSF-Q severity scores. Given the reduced number of prevalent health states obtained for the generic instruments (especially for EQ-5D-3 L) the question arises whether some characteristic or “natural” disease-related health states could be identified for each generic instrument, discarding other comorbidity-influenced health states. From a nosological point of view, it looks quite tempting to think that GERD would not entail a high deterioration in mobility, but it could be the case that bed-ridden people might very likely develop GERD. One possible way to minimize the impact of comorbidities, when measuring specific health conditions with a generic instrument, would be to use a set of instructions demanding that the patient assess his or her overall health condition while thinking only of his or her specific disease.

## Limitations

The present study has been carried out with a Spanish population, and we cannot ensure that other cultural or eating habits would not distort our results.

## Conclusions

In the present study two methods are presented allowing the mapping of specific GERD-severity scores obtained by use of the GSF-Q, onto generic HRQoL values, as measured by the SF-36 and EQ-5D-3 L instruments. In both cases, the cubic model attains best adjustment.

Mapping is an approach that enables utilities to be predicted for the calculation of quality-adjusted life-years when no preference-based information has been elicited what will allow to elaborate health economic evaluations in a simpler way, since it is not necessary to have data of no preference-based instruments. The results of this study will allow to carry out economic evaluations in the world of gastroesophageal reflux disease which will help in the future when it is necessary to make decisions with new alternatives that arrive at the market.

## Abbreviations

BMI: Body Mass Index
CFI: Comparative fit index
CIED: Cardiac implantable device
EQ-5D-3 L: EuroQol 5 Dimensions 3 Levels
GERD: Gastroesophageal Reflux Disease
GOF: Goodness-of-fit
GSF-Q: Gastrointestinal Short Form Questionnaire
H2RA: H_2_ Receptor Antagonist
HRQoL: Health Related Quality of Life
M: Mean
MAE: Mean absolute error
MAPE: Mean percentage absolute error
MAUF: multi-attribute utility function
MOS SF-36: Medical Outcome Survey Sort Form – 36 items
p: significance level
PBM: Preference-based measure
PPI: Proton pump inhibitors
PROM: patient-reported-outcome measurement
Q1: 1st Quartile
Q3: 3rd Quartile
R^2^: R-square GOF statistic
RMSEA: Root mean square error of approximation
SD: Standard Deviation
SF-6D: Medical Outcome Survey Sort Form 6 dimensions
TLI: Tucker-Lewis fit index
VAS: Visual Analogue Scale
